# Are the Motives for Food Choices Different in Orthorexia Nervosa and Healthy Orthorexia?

**DOI:** 10.3390/nu11030697

**Published:** 2019-03-25

**Authors:** Julia Depa, Juan Ramón Barrada, María Roncero

**Affiliations:** 1 Department of Nutritional, Food and Consumer Sciences, Fulda University of Applied Sciences, 36037 Fulda, Germany; julia.depa@pg.hs-fulda.de; 2 Departamento de Psicología y Sociología, Universidad de Zaragoza, 44003 Teruel, Spain; 3 Departamento de Personalidad, Evaluación y Tratamientos Psicológicos, Universitat de València, 46010 Valencia, Spain; maria.roncero@uv.es

**Keywords:** orthorexia nervosa, healthy orthorexia, eating disorders, disordered eating, food choice motives

## Abstract

Recent research points to the bidimensional nature of orthorexia, with one dimension related to interest in healthy eating (healthy orthorexia) and another dimension related to a pathological preoccupation with eating healthily (orthorexia nervosa). Research was needed to provide further support for this differentiation. We examined the food-choice motives related to both aspects of orthorexia. Participants were 460 students from a Spanish university who completed the Teruel Orthorexia Scale and the Food Choice Questionnaire. By means of structural equation modeling, we analyzed the relationship between orthorexia, food-choice motives, gender, body mass index, and age. The motives predicting food choices in orthorexia nervosa and healthy orthorexia were quite different. In the case of orthorexia nervosa, the main motive was weight control, with sensorial appeal and affect regulation also showing significant associations. For healthy orthorexia, the main motive was health content, with sensorial appeal and price also showing significant associations. This supports the hypothesis that orthorexia nervosa is associated with maladaptive eating behavior motived more by weight control than by health concerns.

## 1. Introduction

More than twenty years ago, Steven Bratman, a practitioner of “alternative medicine” [1], coined the term “orthorexia nervosa” (OrNe) [2]: when the intention to eat healthily becomes an unhealthy obsession. Affected people categorize food into “right and healthy” and “wrong and unhealthy”. This eating behavior, which can be considered disturbed, focuses on a perfect, pure, and healthy diet to positively influence one’s health [2]. People with a high level of OrNe value purity of food above all else, including the deleterious health effects of such a diet [3]. After this initial description, several case reports followed. According to these reports, the desire to be thin did not seem to be the main motive, but rather the desire to eat healthily [4,5,6,7,8].

Over time, the diet becomes more rigid, and eating foods categorized as “unhealthy” causes feelings of guilt, fear of becoming ill, and self-punishment behaviors, such as following an even more restricted diet [4,8]. It was also reported that the person tries to convince other people to follow the same diet [4,8]. Suffering from OrNe can result in social isolation, impaired quality of life, malnutrition, and extreme weight loss [4,5,6,7,8].

OrNe has been positively associated with disturbed eating behavior or symptoms of disturbed eating behavior and obsessiveness [9,10]. There has been debate about whether OrNe is a distinct eating disorder, a variant of existing eating disorders (anorexia nervosa or bulimia nervosa) [11,12], a coping strategy of patients with an eating disorder [9,13,14,15], or just a lifestyle [16,17]. To date, OrNe has not been recognized in the Diagnostic and Statistical Manual [18], and, therefore, no official diagnostic criteria exists [9,11].

Different studies have tried to link OrNe to various personal characteristics. Most studies have shown no association or a small one between OrNe and body mass index (BMI) [3,10,19], although a positive relationship has also been reported [20]. There has also been research on the association between OrNe and the area of university studies, with mixed results. Students of nutrition science, who are more interested in health and health food choices and show more dietary restrained eating behavior, have shown higher levels of OrNe than students without a nutritional background in some studies [21], but not in others [22,23]. Apparently, gender is unrelated or minimally related to OrNe [20]. 

Research in this field has been hindered, until recently, by a lack of valid measurement tools distinguishing between healthy and pathological eating behaviors [24,25]. The measuring tool used most often to assess OrNe is the ORTO-15 [26], which is based on Bratman’s descriptions and the Bratman Orthorexia Test (BOT) [8,11]. The BOT is an informal self-assessment measure that has not been validated and has unknown psychometric properties [3,26]. Thus, the ORTO-15 has some important limitations related to its psychometric characteristics: Instability in its internal structure and internal consistency [22], doubts about the scoring scheme [25] and the interpretation of the questionnaire scores [27,28,29], and, perhaps more importantly, limitations in content validity [25,30]. Negative reliabilities have even been reported [27]. From our point of view, the psychometric limitations of the ORTO-15 are so important that knowledge about OrNe gained through this questionnaire should be viewed with extreme caution. These metric problems led to the development of other tools to assess OrNe, such as the Eating Habits Questionnaire (EHQ) [21] and the Düsseldorfer Orthorexia Scale (DOS) [31]. The psychometric properties of these questionnaires have been tested, showing good internal consistency, good test-retest reliability, and good construct validity [20,21,31,32,33]. 

A promising new instrument is the Teruel Orthorexia Scale (TOS) [10]. A key advantage of the TOS is that it distinguishes between healthy orthorexia (HeOr) and OrNe. From this point of view, orthorexia is a broader construct than OrNe, and the positive dimension of healthy eating should also be considered. In this regard, orthorexia would be similar to other constructs that make it possible to differentiate between a healthy/protective dimension and a pathological dimension, such as adaptive and maladaptive perfectionism [34], harmonious and obsessive passion [35], or isolated and compulsive pornography consumption [36]. 

Items in the HeOr factor assess a healthy interest with diet, healthy behavior with regard to diet, and eating healthily as part of one’s identity. After partitioning the OrNe effect, HeOr does not correlate—or even presents negative correlations—with psychopathology measures, namely disordered eating, perfectionism, and obsessive-compulsive behavior. By contrast, items in the OrNe factor assess excessive preoccupation with a healthy diet, punishment when violating personal rules, and a negative impact on social life due to eating restrictions. This factor correlates with psychopathology measures. Thus, OrNe and HeOr do not function as a continuum from people who do not care at all about eating healthily, followed by people who eat healthily (HeOr), and, finally, those who care excessively (OrNe). In fact, the correlation between HeOr and OrNe was only 0.43 [10]. The availability of better instruments like the TOS has been helpful, for instance, to show that, contrary to speculations, trying to convince people to follow healthy eating habits should not be considered an element of OrNe, but rather an indicator of HeOr.

It is important to assess the valid distinction between OrNe—a pathological eating behavior—and HeOr, in order to avoid producing undifferentiated scientific results and misclassification of investigated study participants as pathological, when they are merely health-conscious. Until now, no studies have investigated the motives associated with OrNe or HeOr. Therefore, the main aim of the present study is to investigate whether there are differences in the association between motives for choosing food in OrNe and HeOr. In doing so, we expect to gain valuable information. First, we can obtain additional evidence that OrNe and HeOr should be considered separate factors, as this proposal is quite recent. Second, we can find out which motives are connected to orthorexia. A previous instrumental step was to test the psychometric properties of the Food Choice Questionnaire [37,38].

Regarding the main objective, we had two hypotheses: The first, that the motives associated with HeOr and OrNe would differ because these eating styles do not lie on the same continuum. Second, that although this was more exploratory, we expected that for OrNe the main motive would be weight control, given the large association between OrNe and restrained eating [10]. OrNe would also be associated with choosing food for affect regulation; this refers to our efforts to influence our valence responses and includes aspects such as coping, emotion regulation, and mood regulation [39]. However, we expected HeOr to be mainly associated with motives related to the healthy composition of the food. 

## 2. Materials and Methods

### 2.1. Participants and Procedure

The present study formed part of a more comprehensive project carried out in a Spanish university to determine the key correlates of orthorexia. Data were collected in December 2016. We approached the participants through the e-mail distribution lists of the University of Zaragoza (Aragón, Spain). Each student registered on the lists whose administrators gave access to the corresponding information received an e-mail with the goal of the study, contact information of the principal investigator, participation conditions, and a link to access the survey. We used Google Forms. Only those who accepted the informed consent could gain access. This procedure was approved by the regional Ethics Review Board for Clinical Research (C.P.-C.I. PI18/340).

The initial sample was made up of 575 participants between 17 and 68 years old (*M* = 23.20, *SD* = 6.52). Four inclusion criteria were employed: (1) Being a resident in Spain (seven participants excluded); (2) currently studying at the university (seven participants excluded); (3) being between 18 and 26 years old, based on criteria from previous studies with university samples [36,40,41] (78 participants excluded); and (4) correctly answering a control question (see below; 23 participants excluded). After applying these criteria, the final sample consisted of 460 participants. Of them, 375 were women (82.0%) and 85 men (18.0%). Mean age was 21.12 years (SD = 2.19). We verified that our dataset contained no duplicate online questionnaires, that is, no pair of participants showed exactly the same responses to all the items.

### 2.2. Instruments

The study battery was administered in Spanish.

#### 2.2.1. Sociodemographic Data

Participants provided information about their gender, age, and education level. They also reported their weight (to the nearest kilogram) and height (to the nearest centimeter).

#### 2.2.2. Teruel Orthorexia Scale (TOS)

This scale [10] assesses orthorexia with two separate dimensions, HeOr (nine items; e.g., “I mainly eat foods that I consider healthy”) and OrNe (eight items; e.g., “Thoughts about healthy eating do not let me concentrate on other tasks”). Responses are provided on a 4-point scale ranging from 0 = strongly disagree to 3 = strongly agree. Scores by dimension were computed as the sum of the item responses. Participants were asked to respond without providing a specific timeframe. With the sample in which the scale was developed, the TOS presented satisfactory internal consistency above 0.80 for both factors, and the test-retest correlation, with a time-lapse of 18 months, was above 0.70 for both factors.

#### 2.2.3. Food Choice Questionnaire (FCQ)

This scale [37] assesses food-choice motives with 36 items. Responses are provided on a 4-point scale ranging from 1 = not at all important to 4 = very important. We used the Spanish version [42]. Scores by dimension were computed as the mean of the item responses. Item wording appears in Table 1. Participants were asked to respond with a normal day in mind.

The internal structure, that is, the number of measured factors and the assignment of the items to the factors, of the FCQ is unclear. Whereas Steptoe et al. [37] identified nine factors, Jáuregui-Lobera and Bolaños Ríos [42], in their final Spanish version, deleted two items and retained seven factors. Fotopoulos et al. [38] changed the response scale to a 7-point scale, shortened the FCQ to a 24-item version, and retained eight factors. We consider that some of these inconsistencies can be attributed to the use of suboptimal psychometric techniques, such as Varimax rotations, unclear criteria to select the number of factors to retain, or inadequate estimation techniques given the categorical nature of the responses [43]. For these reasons, we started with the full version of the FCQ and performed a new study of its internal structure with more adequate psychometric techniques. Given that the specific number of assessed dimensions is unclear, as is the distribution of items per dimension, we cannot list the factors or provide examples of items for each factor.

#### 2.2.4. Control Question

Embedded in the FCQ as its 37th item, we introduced an item asking the participants to provide a specific response: “Not at all important”. By doing this, we checked whether the participants paid enough attention to the wording of the items. Those participants responding with an option different from the one requested could be considered distracted. 

### 2.3. Data Analyses

Given the inconsistencies mentioned above regarding the FCQ internal structure, as a preliminary analysis we tested the dimensional structure of this questionnaire with an exploratory structural equation model (ESEM) [44]. As the number of factors to be extracted from the FCQ was uncertain, in order to determine the numbers of dimensions to be retained, we used parallel analysis [45] and considered the theoretical interpretability of the solutions, factor simplicity, and loading sizes. In order to develop a usable FCQ with a more clearly identified and stable factor structure, we selected the items that clearly loaded in a single factor. From the selected factor solution with the full FCQ, items were dropped if (a) loadings in all the factors were below 0.50, or (b) more than a single loading was above 0.30, with the restriction that at least three items were assigned per dimension. A similar approach has been applied in the refinement of other scales assessing motives [46]. Henceforth, we will refer to these selected items as the Short Food Choice Questionnaire (Short FCQ). Finally, means, standard deviations, and reliabilities (Cronbach’s α) of the total scores by dimension were computed.

In order to examine the relationships between OrNe and HeOr and the FCQ factors and sociodemographic information (gender, BMI, and age), two sets of analyses were performed. First, we analyzed the factor structure of the items on the TOS and the FCQ together, with TOS scores and FCQ defining two separate sets of factors, and including the sociodemographic variables in the model. Thus, we could compute the correlations between the orthorexia dimensions, the food-choice dimensions, and the sociodemographic variables at the latent level. Second, we computed a structural equation model where the two orthorexia dimensions were predicted by the different food-choice motives and gender, BMI, and age. 

Goodness of fit of all the derived models was assessed with the common cut-off values for the fit indices [47]: Comparative fit index (CFI) and Tucker–Lewis index (TLI) with values greater than 0.95 and root mean square error of approximation (RMSEA) below 0.06 indicated a satisfactory fit. For all the models, the M*Plus* diagonally weighted least squares estimator (WLSMV) was used. By using this estimator, we were able to maintain the categorical nature of the responses [48]. For all the factor models, we interpreted the standardized solution (STDYX solution in M*Plus*).

All the latent models were estimated with M*plus* 7.4 [49]. The rest of the analyses were performed with *R* 3.5.1 (R Foundation for Statistical Computing, Vienna, Austria) [50]. We used the packages *psych* version 1.6.12 [51] and *MplusAutomation* version 0.7 [52]. No missing data were present in our database. The open database and code files for these analyses are available at the Open Science Framework repository (https://osf.io/kagxy/).

## 3. Results

### 3.1. Psychometric Properties of FCQ

**Figure 1:**
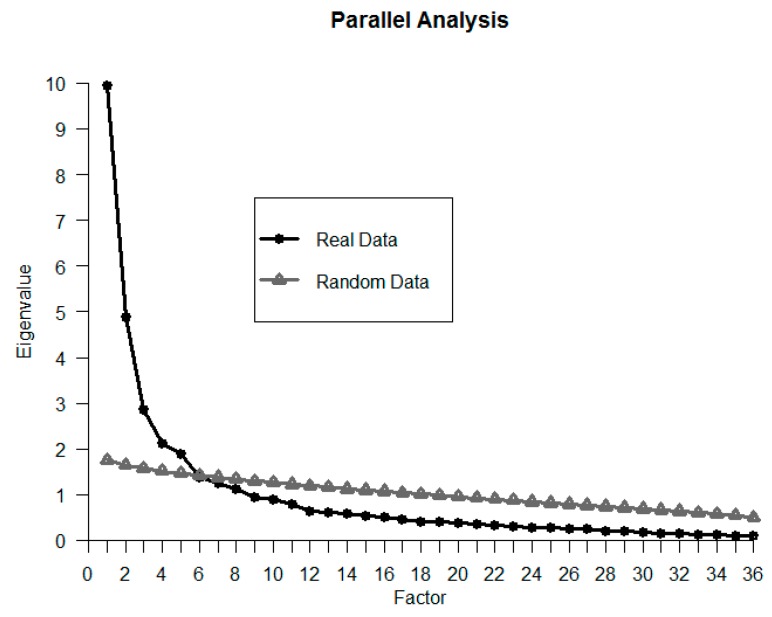
Parallel analysis of the Food Choice Questionnaire (FCQ) responses.

Prior to analysis, the reasons for food choices associated with OrNe and HeOr and the psychometric properties of the FCQ were examined. First, the internal structure was tested. A parallel analysis was performed in order to provide information about the number of factors to retain (Figure 1), suggesting the extraction of five factors. Then, different models were tested with ESEM. Goodness of fit indices are shown in Table 1. The five-factor solution (M1 in Table 1) provided a poor fit (CFI = 0.911, TLI = 0.878, RMSEA = 0.073). More importantly, with the proposed cutoff values for maintaining the items on the Short FCQ, no single item would be retained in the fifth factor because all items presented cross-loadings over 0.42. The six-factor solution (M2) presented the same problems: (a) poor fit (CFI = 0.932, TLI = 0.900, RMSEA = 0.066), and (b) no item retained in the last factor, with all cross-loadings over 0.31. The seven-factor solution still presented a poor fit (CFI = 0.932, TLI = 0.913, RMSEA = 0.061), but it could be theoretically interpreted, and all the factors could have items clearly assigned to them (see factor loadings in Table 2). The seven factors (M3) were labelled Weight Control (e.g., “Is low in calories”), Sensorial Appeal (e.g., “Tastes good”), Convenience (e.g., “Is easy to prepare”), Health Content (e.g., “Keeps me healthy”), Price (e.g., “Is cheap”), Affect Regulation (e.g., “Helps me relax”), and Sociopolitical (e.g., “Comes from countries I approve of politically”). In order to have at least three items per dimension, Item 12 (“Is good value for money”) was kept in the Short FCQ to assess the Price factor, although it presented a loading above 0.30 in the Sensorial Appeal factor. The proposed Short FCQ was composed of 25 items, 11 less than the initial version. The selected items are shown in Table 2.

Model fit for the seven-factor solution of the Short FCQ (M4) was considered satisfactory (CFI = 0.979, TLI = 0.956, RMSEA = 0.053). The theoretical interpretation of this model was clear, with the same seven factors already listed. Moreover, as Table 3 shows, all the reliabilities of the total scores were adequate, in the range 0.76–0.87. The most endorsed motive for food choice was Sensory Appeal (*M* = 3.08), whereas the least relevant motives were Sociopolitical (*M* = 1.99).

### 3.2. Food Choice in Orthorexia Nervosa and Healthy Orthorexia

The first step in examining the relationship between OrNe and HeOr and the FCQ factors and sociodemographic information was to analyze the factor structure of the items on the TOS and the Short FCQ and gender, BMI, and age at the same time (see Table 3 for descriptive statistics and reliabilities). Results (M5) showed that model fit was adequate (CFI = 0.966, TLI = 0.956, RMSEA = 0.036). The correlations between factors involving the orthorexia factors are shown in Table 3. OrNe showed a high positive correlation with Weight Control (*r* = 0.62), and to a lesser extent with Health Content (*r* = 0.22), Affect Regulation (*r* = 0.19) and Sociopolitical motives (*r* = 0.14). OrNe showed a negative correlation with Sensory Appeal (*r* = −0.22). Regarding HeOr, the pattern of associations was different. The highest association was observed with Health Content (*r* = 0.69), followed by Weight Control (*r* = 0.40) and Sociopolitical reasons (*r* = 0.35). Although lower, HeOr presented significant associations in a negative direction with Sensory Appeal, Price, and Convenience (*r* in the range of −0.19 to −0.14). Women and men did not show statistically significant differences in their orthorexia scores. Higher BMI values were related to higher OrNe (*r* = 0.11) and lower HeOr (*r* = −0.15). Age presented a positive correlation with HeOr (*r* = 0.11) and a negative correlation with OrNe (*r* = −0.20).

**Figure 2:**
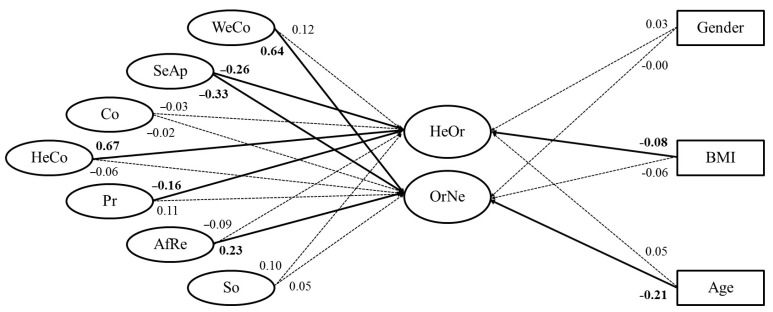
Structural model of the relationships between the seven food-choice motives and orthorexia. Solid lines correspond to statistically significant coefficients; dashed lines correspond to non-statistically significant coefficients. Gender is coded with a dummy variable where 0 = women and 1 = men. Ovals correspond to latent variables. Rectangles correspond to manifest variables. Bold values correspond to statistically significant coefficients, *p* < 0.05. Positive values correspond to positive coefficients in the structural model; negative values correspond to negative coefficients. WeCo = Weight Control; SeAp = Sensorial Appeal; Co = Convenience, HeCo = Health Content; Pr = Price; AfRe = Affect Regulation; So = Sociopolitical; HeOr = Healthy Orthorexia; OrNe = Orthorexia Nervosa.

In order to study whether the orthorexia dimensions were predicted by the different food-choice motives and sociodemographic variables, a structural model of the TOS, the Short FCQ, gender, BMI, and age was performed. This model is equivalent in terms of fit to M5. This model is depicted in Figure 2. We will only comment on the statistically significant coefficients above 0.15. HeOr was positively related to Health Content (β = 0.67) and negatively related to Sensory Appeal (β = −0.26) and Price (β = −0.16). OrNe was positively related to Weight Control (β = 0.64) and Affect Regulation (β = 0.23), and negatively related to Sensory Appeal (β = −0.33) and age (β = −0.21). With this model, 64.5% of the variance in HeOr was explained by food-choice motives, and 54.6% of the variance in OrNe.

## 4. Discussion

The main objective was to explore whether motives for food choices differed between OrNe and HeOr. Once the internal structure of the FCQ had been clarified and its psychometric properties had been tested, the association between motives for food choice and OrNe and HeOr were analyzed. The overall result was that the pattern of associations was different between HeOr and OrNe, confirming our first hypothesis. However, it is important to note that the pattern of associations found in bivariate correlations was different from the pattern found with the structural model. As we think the multivariate SEM model is a better approach to detect the key associations—it allowed us to partition out the influence of other predictors—we will mainly discuss these results. 

The only predictor in the SEM model that was shared by OrNe and HeOr was sensory appeal, with a low–medium negative relation in both cases. This result is not surprising, given the descriptions of HeOr and OrNe [9,11,12,17]. In both the pathological and healthy dimensions of orthorexia, the person would not succumb to temptation due to a food’s good appearance. As mentioned above, with the exception of sensory appeal, the pattern of predictors for OrNe and HeOr diverged. 

For OrNe, the motive presenting the highest (and positive) relation was weight control, as we predicted in our second hypothesis, based on the association found between OrNe and eating disorders. However, this result is not congruent with the case report descriptions of affected people, who report that their eating behavior was mainly motivated by wanting to be healthy and not by weight control [4,5,7]. This result can be interpreted in two ways: It is possible that the person has the desire to control his/her weight in order to avoid the health problems associated with being overweight and obesity. The weight control factor contains items such as “is low in fat”, which can be considered a characteristic of a healthy diet [53]. This is not strange, given the campaigns carried out worldwide to warn people about the complications of being overweight and obesity. In fact, the World Health Organization [53] warns that obesity is one of the main worldwide risk factors for death. However, if this interpretation is correct, the health content motive would also arise as a predictor of OrNe, but surprisingly, health content presented a negligible and statistically non-significant relationship with OrNe. Another interpretation would be that OrNe is a camouflaged eating disorder. This position is shared by many health professionals [54]. Segura-Garcia et al. [14] reported that eating disorder patients showed increased orthorexia tendencies at the end of therapy compared to the beginning. Diverse cross-sectional studies repeatedly showed an association between OrNe and pathological eating behavior [10,19,55,56,57]. All in all, more studies examining the connection between OrNe and disordered eating behavior are needed to understand this behavior for treatment and health promotion. Even so, cultural studies looking at Western-oriented health and weight ideals could help to understand the connection.

Continuing with OrNe, the food-choice motive with the second highest (and positive) association, with a low–medium relationship, was affect regulation, supporting our second hypothesis. This result is not surprising because health professionals have stated that orthorexic eating behavior helps affected people to control emotions such as fear [56]. Moreover, it has been demonstrated that difficulties with emotional regulation play an important role in the etiology and maintenance of several mental disorders, such as eating disorders [58]. Eating disorder patients use restriction and purges as a way to cope with negative emotions and extreme positive emotions [59,60].

In the case of HeOr, the food-choice motive with the highest (and positive) relationship was health content. As we have seen, this motive was not a predictor in the case of OrNe, supporting the idea that OrNe is qualitatively different from HeOr, which represents an adaptive interest in healthy food [10]. Price predicted HeOr with a low and negative association. This result, as in the case of sensory appeal, is not surprising. People who are interested in buying organic food are willing to pay more for it because they are aware that the cost of producing organic food is greater, as consumer studies reveal [61,62].

We also tested the association between some sociodemographic variables and both dimensions of orthorexia. Gender did not show significant coefficients with either dimension of orthorexia. BMI presented very small negative associations with OrNe (non-significant) and HeOr (statistically significant) [10]. Age showed a negative relationship with OrNe.

The main strengths of this study are the questionnaires used and the analytical approach. First, the FCQ incorporates a wide range of food-choice motives. By testing its internal structure, we were able to produce a Short FCQ (31% shorter) with a clearer structure, but adequate reliability. Second, the TOS distinguishes between maladaptive and healthy eating behavior. However, it should be mentioned that the development of the TOS is in its early stages. Validations in non-Spanish and non-university student samples are required. Third, relying on latent variables modeled with an ESEM approach, rather than manifest variables, reduces the risk of the results being affected by measurement error and of the items tapping two dimensions at the same time [45,63,64]. 

Several limitations should be noted. The study sample (only students) means that our results are not representative of the general population. We cannot calibrate how important the problem of representativeness is. For instance, it is possible that university students are not used to buying their own food: many university students in Spain live with their parents. In addition, by having the questionnaires filled out online rather than in a controlled environment, it is uncertain whether people understood all the questions. Given the high reliability found, apparently this was not a problem, although we cannot completely rule out this possibility. Our sample was mainly female. This problem has been found in other studies with the same sampling scheme in the same university [36,41,63]. In order to reduce potential biases, we have included gender as a covariate in our analysis. Additionally, we have only tapped some of the motives that could lead to orthorexic tendencies. Further motives for OrNe are having control over life, following spirituality motives, or reaching perfection [7]. As the FCQ does not measure these motives or any disturbed motives, OrNe has to be explored by looking for associations with the aforementioned motives and disturbed motives. OrNe is more common among vegetarians and vegans, compared to people who are not following a special diet [23,65,66]. Therefore, animal-related motives, which in 90% of vegetarians are the main motives for their diet [67], should be explored. Lastly, the TOS and our results are culture-specific; so far, they have only been investigated with Spanish participants. Studies validating the TOS and motives in different cultures are necessary. 

## 5. Conclusions

Our results show that the main measured motives for OrNe and HeOr are quite different. In the case of OrNe, the main motive is weight control, whereas for HeOr it was health content. This result supports the hypothesis that OrNe, as measured by the TOS and other measurement tools, is associated with maladaptive eating behavior motived more by weight control than health reasons. Further studies should analyze the association between OrNe and eating disorder symptoms. 

## Figures and Tables

**Table 1:** Goodness of fit indices for the different models.

Models	*χ* ^2^	*df*	CFI	TLI	RMSEA
M1. FCQ 5 Factors	1573.9	460	0.911	0.878	0.073
M2. FCQ 6 Factors	1283.4	429	0.932	0.900	0.066
M3. FCQ 7 Factors	1086.8	399	0.945	0.913	0.061
M4. Short FCQ	333.1	146	0.979	0.956	0.053
M5. TOS & Short FCQ & Gender & BMI & Age	1214.8	759	0.966	0.956	0.036

*χ*^2^ = Pearson chi-square test; df = degrees of freedom; TLI = Tucker–Lewis index; CFI = comparative fit index; RMSEA = root mean square error of approximation; TOS = Teruel Orthorexia Scale; FCQ = Food Choice Questionnaire. All *p*-values for the *χ*^2^ test were <0.001.

**Table 2:** Item loadings in the initial FCQ and Short FCQ and among factor correlations.

	**Initial FCQ Version**								**Short FCQ Version**						
	**Item Loadings**								**Item Loadings**						
	**WeCo**	**SeAp**	**Co**	**HeCo**	**AfRe**	**So**	**Pr**		**WeCo**	**SeAp**	**Co**	**HeCo**	**Pr**	**AfRe**	**So**
01. Is easy to prepare	0.05	−0.13	**0.84**	−0.01	−0.02	0.03	0.02		0.01	−0.11	**0.87**	0.00	−0.02	−0.02	0.00
* 02. Contains no additives	0.26	−0.06	−0.19	**0.40**	−0.04	**0.36**	0.12		–––	–––	–––	–––	–––	–––	–––
03. Is low in calories	**0.90**	−0.05	0.02	0.06	0.02	0.00	0.00		**0.91**	−0.08	−0.02	0.04	0.03	0.01	0.02
04. Tastes good	−0.02	**0.74**	0.02	0.07	−0.06	−0.15	0.02		0.03	**0.75**	0.01	0.09	0.04	−0.08	−0.15
* 05. Contains natural ingredients	0.10	0.05	−0.09	**0.50**	−0.07	**0.35**	0.06		–––	–––	–––	–––	–––	–––	–––
06. Is not expensive	0.02	0.03	0.00	−0.13	0.04	0.03	**0.85**		0.08	0.00	−0.06	−0.13	**0.88**	−0.01	0.02
07. Is low in fat	**0.81**	0.06	0.03	0.10	−0.03	0.02	0.02		**0.82**	0.04	0.01	0.08	0.03	−0.03	0.03
* 08. Is familiar	0.03	**0.35**	−0.10	0.13	0.15	−0.03	−0.05		–––	–––	–––	–––	–––	–––	–––
* 09. Is high in fiber and roughage	**0.32**	0.01	0.00	**0.45**	0.06	0.17	−0.06		–––	–––	–––	–––	–––	–––	–––
10. Is nutritious	0.02	0.17	−0.02	**0.70**	−0.13	0.05	−0.05		0.01	0.13	−0.02	**0.75**	−0.04	−0.07	0.02
* 11. Is easily available in shops and supermarkets	0.11	**0.36**	**0.41**	0.02	−0.20	−0.01	0.21		–––	–––	–––	–––	–––	–––	–––
12. Is good value for money	0.03	**0.32**	0.15	0.04	−0.14	0.05	**0.57**		0.03	0.18	0.05	0.10	**0.63**	−0.05	0.03
* 13. Cheers me up	0.09	**0.45**	−0.04	−0.08	**0.44**	−0.02	0.10		–––	–––	–––	–––	–––	–––	–––
14. Smells nice	0.01	**0.78**	−0.11	−0.19	0.22	0.08	0.05		−0.02	**0.72**	−0.11	−0.08	0.08	0.23	0.05
15. Can be cooked very simply	0.07	0.03	**0.86**	−0.05	0.09	0.01	0.05		0.02	0.03	**0.86**	−0.03	0.03	0.09	−0.02
16. Helps me cope with stress	0.25	0.06	0.09	−0.09	**0.72**	0.09	0.00		0.20	0.06	0.11	−0.09	−0.02	**0.70**	0.07
17. Helps me control my weight	**0.84**	0.02	0.02	0.03	0.17	−0.07	−0.05		**0.84**	0.00	0.00	0.00	−0.04	0.18	−0.05
18. Has a pleasant texture	0.01	**0.66**	0.04	−0.04	0.15	0.05	−0.05		0.06	**0.71**	0.02	−0.05	−0.04	0.14	0.07
19. Is packaged in an environmentally friendly way	−0.01	0.08	0.04	0.08	−0.01	**0.63**	0.08		0.04	0.11	0.03	0.06	0.06	−0.01	**0.63**
20. Comes from countries I approve of politically	−0.03	−0.02	0.07	−0.02	0.00	**0.94**	−0.05		0.04	−0.05	0.02	−0.01	−0.01	−0.02	**0.94**
* 21. Is like the food I ate when I was a child	−0.07	0.22	0.10	0.03	0.24	0.21	−0.05		–––	–––	–––	–––	–––	–––	–––
22. Contains a lot of vitamins and minerals	0.04	−0.01	0.03	**0.69**	0.05	0.19	−0.04		0.01	−0.09	−0.02	**0.74**	0.02	0.13	0.15
* 23. Contains no artificial ingredients	0.16	0.00	−0.26	**0.46**	0.04	**0.43**	0.12		–––	–––	–––	–––	–––	–––	–––
24. Keeps me awake/alert	0.03	−0.05	−0.06	0.24	**0.65**	−0.04	0.13		0.03	−0.01	−0.01	0.19	0.09	**0.62**	−0.04
25. Looks nice	−0.11	**0.53**	0.05	0.13	0.28	−0.11	−0.02		−0.10	**0.55**	0.06	0.13	−0.03	**0.31**	−0.13
26. Helps me relax	0.03	0.04	−0.05	0.12	**0.77**	0.07	0.04		−0.03	0.02	−0.03	0.10	−0.01	**0.88**	0.01
27. Is high in protein	0.08	−0.06	0.03	**0.50**	0.25	0.06	−0.02		0.03	−0.14	0.00	**0.57**	0.02	**0.34**	0.00
28. Takes no time to prepare	0.01	−0.08	**0.86**	−0.01	0.03	−0.03	0.06		0.01	−0.01	**0.87**	−0.03	0.02	−0.02	−0.01
29. Keeps me healthy	0.12	0.01	0.02	**0.82**	−0.02	−0.07	0.01		0.20	0.04	0.03	**0.74**	−0.02	−0.03	−0.02
30. Is good for my skin/teeth/hair/nails, etc.	−0.05	0.01	−0.02	**0.67**	0.24	0.10	0.07		0.04	0.05	−0.01	**0.59**	0.05	0.21	0.14
* 31. Makes me feel good	−0.04	0.16	0.06	**0.55**	**0.48**	−0.22	0.01		–––	–––	–––	–––	–––	–––	–––
32. Has the country of origin clearly marked	−0.16	0.02	0.03	0.21	0.05	**0.78**	−0.07		−0.11	0.00	−0.02	0.22	−0.03	0.03	**0.78**
* 33. Is what I usually eat	−0.03	0.20	0.20	0.17	0.14	−0.03	−0.12		–––	–––	–––	–––	–––	–––	–––
* 34. Helps me to cope with life	−0.01	−0.03	0.04	**0.30**	**0.71**	0.03	−0.09		–––	–––	–––	–––	–––	–––	–––
35. Can be bought in shops close to where I live or work	−0.12	0.18	**0.54**	0.17	0.01	0.04	0.22		−0.06	0.14	**0.48**	0.11	0.26	0.01	0.08
36. Is cheap	−0.13	−0.01	0.14	0.02	0.11	−0.07	**0.88**		−0.08	−0.03	0.06	0.00	**0.94**	0.07	−0.07
	Interfactor Correlations								Interfactor Correlations						
	WeCo	SeAp	Co	HeCo	AfRe	So	Pr		WeCo	SeAp	Co	HeCo	Pr	AfRe	So
WeCo								WeCo							
SeAp	0.03							SeAp	−0.01						
Co	0.02	0.20						Co	0.06	0.21					
HeCo	0.45	0.27	0.03					HeCo	0.44	0.21	0.02				
AfRe	0.09	0.35	0.21	0.22				Pr	0.10	0.34	0.45	0.17			
So	0.22	0.11	0.00	0.42	0.22			AfRe	0.16	0.32	0.20	0.25	0.18		
Pr	0.14	0.30	0.36	0.17	0.11	0.12		So	0.17	0.10	0.02	0.41	0.13	0.26	

FCQ = Food Choice Questionnaire; WeCo = Weight Control; SeAp = Sensorial Appeal; Co = Convenience, HeCo = Health Content; Pr = Price; AfRe = Affect Regulation; So = Sociopolitical. Item wording with an asterisk (*) indicates that the item is not included in the Short FCQ. Loadings in bold indicate unsigned loadings above 0.30. Shaded cells indicate the factor where the item was assigned in the short FCQ. Underlined loadings indicate cross-loadings above 0.30 in the Short FCQ.

**Table 3:** Correlations and descriptive statistics of TOS and FCQ.

	Correlations					Descriptive Statistics		
	HeOr	OrNe	Gender	BMI	Age	Mean	SD	Alpha
TOS Healthy Orthorexia			0.03	**−0.15**	**0.11**	12.71	5.26	0.85
TOS Orthorexia Nervosa	**0.35**		−0.09	**0.11**	**−0.20**	4.32	4.05	0.84
Short FCQ Weight Control	**0.40**	**0.62**	**−0.18**	**0.21**	0.07	2.34	0.89	0.87
Short FCQ Sensorial Appeal	**−0.19**	**−0.22**	**−0.22**	-0.10	0.05	3.08	0.64	0.76
Short FCQ Convenience	**−0.14**	0.04	0.01	0.02	0.01	2.68	0.74	0.84
Short FCQ Health Content	**0.69**	**0.22**	−0.04	**−0.14**	0.08	2.77	0.70	0.82
Short FCQ Price	**−0.15**	0.07	0.09	**0.13**	0.01	2.89	0.68	0.81
Short FCQ Affect Regulation	−0.09	**0.19**	**−0.14**	0.01	−0.07	2.11	0.82	0.77
Short FCQ Sociopolitical	**0.35**	**0.14**	−0.01	−0.07	0.08	1.99	0.84	0.80

TOS = Teruel Orthorexia Scale; FCQ = Food Choice Questionnaire. Gender is coded with a dummy variable where 0 = women and 1 = men. Bold values correspond to statistically significant results, *p* < 0.05.
